# Deep odontogenic infections—identifying risk factors for nosocomial pneumonia

**DOI:** 10.1007/s00784-020-03500-4

**Published:** 2020-08-13

**Authors:** Niina Rautaporras, Jussi Furuholm, Johanna Uittamo, Mikko Saloniemi, Tuukka Puolakka, Johanna Snäll

**Affiliations:** 1 Department of Oral and Maxillofacial Diseases, University of Helsinki and Helsinki University Hospital, P.O. Box 220, (Haartmaninkatu 4E), FI-00029 HUH Helsinki, Finland; 2 Department of Emergency Medicine and Services, University of Helsinki and Helsinki University Hospital, Helsinki, Finland; 3 Department of Anaesthesiology and Intensive Care Medicine, University of Helsinki and Helsinki University Hospital, Helsinki, Finland

**Keywords:** Odontogenic infection, Dental abscess, Deep neck infection, Nosocomial pneumonia, Risk factors

## Abstract

**Objectives:**

To evaluate occurrence and risk factors for pneumonia in patients with deep odontogenic infection (OI).

**Materials and methods:**

All patients treated for deep OIs and requiring intensive care and mechanical ventilation were included. The outcome variable was diagnosis of nosocomial pneumonia. Primary predictor variables were re-intubation and duration of mechanical ventilation. The secondary predictor variable was length of hospital stay (LOHS). The explanatory variables were gender, age, current smoking, current heavy alcohol and/or drug use, diabetes, and chronic pulmonary disease.

**Results:**

Ninety-two patients were included in the analyses. Pneumonia was detected in 14 patients (15%). It was diagnosed on postoperative day 2 to 6 (median 3 days, mean 3 days) after primary infection care. Duration of mechanical ventilation (*p* = 0.028) and LOHS (*p* = 0.002) correlated significantly with occurrence of pneumonia. In addition, re-intubation (*p* = 0.004) was found to be significantly associated with pneumonia; however, pneumonia was detected in 75% of these patients prior to re-intubation. Two patients (2%) died during intensive care unit stay, and both had diagnosed nosocomial pneumonia. Smoking correlated significantly with pneumonia (*p* = 0.011).

**Conclusion:**

Secondary pneumonia due to deep OI is associated with prolonged hospital care and can predict the risk of death. Duration of mechanical ventilation should be reduced with prompt and adequate OI treatment, whenever possible. Smokers with deep OI have a significantly higher risk than non-smokers of developing pneumonia.

**Clinical relevance:**

Nosocomial pneumonia is a considerable problem in OI patients with lengthy mechanical ventilation. Prompt and comprehensive OI care is required to reduce these risk factors.

## Introduction

Hospitalized patients with odontogenic infections (OIs) are at risk for life-threatening conditions and infection complications. These severe infections can lead to death despite accurate and multidisciplinary care [1–3]. Elderly patients [3, 4] and patients with underlying disease [5, 6] are more susceptible to complicated infections. Generalized infection of dental origin can cause severe sepsis [1, 2] and spread to distant organs, causing disseminated infections such as endocarditis [5], mediastinitis [1, 2, 7–9], pleural emphysema [7], meningitis [10], subdural emphysema [10], brain abscess [11], necrotizing fasciitis [9, 12], and thrombophlebitis of the internal jugular vein (Lemierre’s syndrome) [13]. Pneumonia is, however, a common secondary infection related to deep OI [2], and when it is acquired during hospitalization, the applicable term is nosocomial pneumonia.

Deep neck infection-associated pneumonia is reported to occur in 1–6% of hospitalized patients [2, 9, 14]. Patients’ general condition affects prognosis, and patients with reduced immune defence have associated respiratory tract infection secondary to OI more often than in other populations [2]. Pneumonia complicates patient care in intensive care units (ICUs) in general; nearly two-thirds (64%) of infections in this patient group are of respiratory origin [15], and as a nosocomial infection, the prevalence of ventilation-associated pneumonia (VAP) is 5–40% [16]. Even though the definition of VAP and reporting standards vary [16, 17], pneumonia in patients with at least 48 h of mechanical ventilation and without preceding pneumonia is the most commonly used definition of VAP. Prolonged ICU stay [16, 18] and re-intubation episodes [19, 20] are known to predispose patients to respiratory infections. Especially extubation failure has been shown to be associated with pneumonia [20]. Thus, unplanned re-intubation and emergency airway management predispose to these infections in general ICU patient populations.

Trismus, oedema, and pus formation in the oropharyngeal region as well as challenging airway management due to narrowed laryngeal space and limited visibility during intubation cause aspiration risk and predispose patients to respiratory infections. We aimed to clarify and evaluate the occurrence and special features of secondary pneumonia in patients treated for deep OI. We hypothesized that occurrence of secondary pneumonia is rather high in OI patients in general and that patient- and treatment-related factors would emerge.

## Materials and methods

### Study design and inclusion criteria

A retrospective cohort study was conducted in the Töölö Hospital Emergency Department of Helsinki University Hospital with a catchment area of 1.6 million inhabitants between 2015 and 2019. Patients who were treated in the oral and maxillofacial surgery’s emergency service for deep OI (i.e., abscess and/or cellulitis of facial and/or neck region from dental origin) confirmed by oral and maxillofacial surgeons and required postoperative mechanical ventilation in the ICU were included in this study. Exclusions comprised patients with unclear infection, patients with other than odontogenic focus, patients with pneumonia at hospital admission, and patients treated without postoperative mechanical ventilation.

### Study variables

The outcome variable was presence of pneumonia, defined as clinically suspected and radiologically confirmed pneumonia during OI treatment in ICU.

Primary predictor variables were re-intubation and duration of mechanical ventilation. The secondary predictor variable was length of hospital stay (LOHS).

Explanatory variables were gender, age, current smoking, current heavy alcohol and/or regular drug use, diabetes (DM), and chronic pulmonary disease. Consumption limits for heavy alcohol use were ≥ 12 doses per week in women and ≥ 23 doses in men, based on anamnestic information. In addition, bacterial stain in tracheal aspirates of pneumonia patients, airway management, need for late tracheostomy, and overall survival rate were recorded. Also, accurate information of pneumonia and administered antibiotic medications were gathered.

### Statistical analysis

We used software package IBM SPSS for Macintosh (version 25.0, IBM Corp., Armonk, NY, USA) for statistical data analyses. For categorical variables, we evaluated differences in associations between the study groups with Pearson’s chi-square test or with Fisher’s exact test if expected values were below 5. We used Student’s *t* test to analyse differences between groups for continuous variables. For multivariate analysis, we selected binomial logistic regression with occurrence of pneumonia as the dependent variable; of the predictors, age was categorized into tertiles, while the rest of the independent variables were dichotomous. Throughout the study, we considered *p* values below 0.05 to be statistically significant.

## Results

**Fig. 1:**
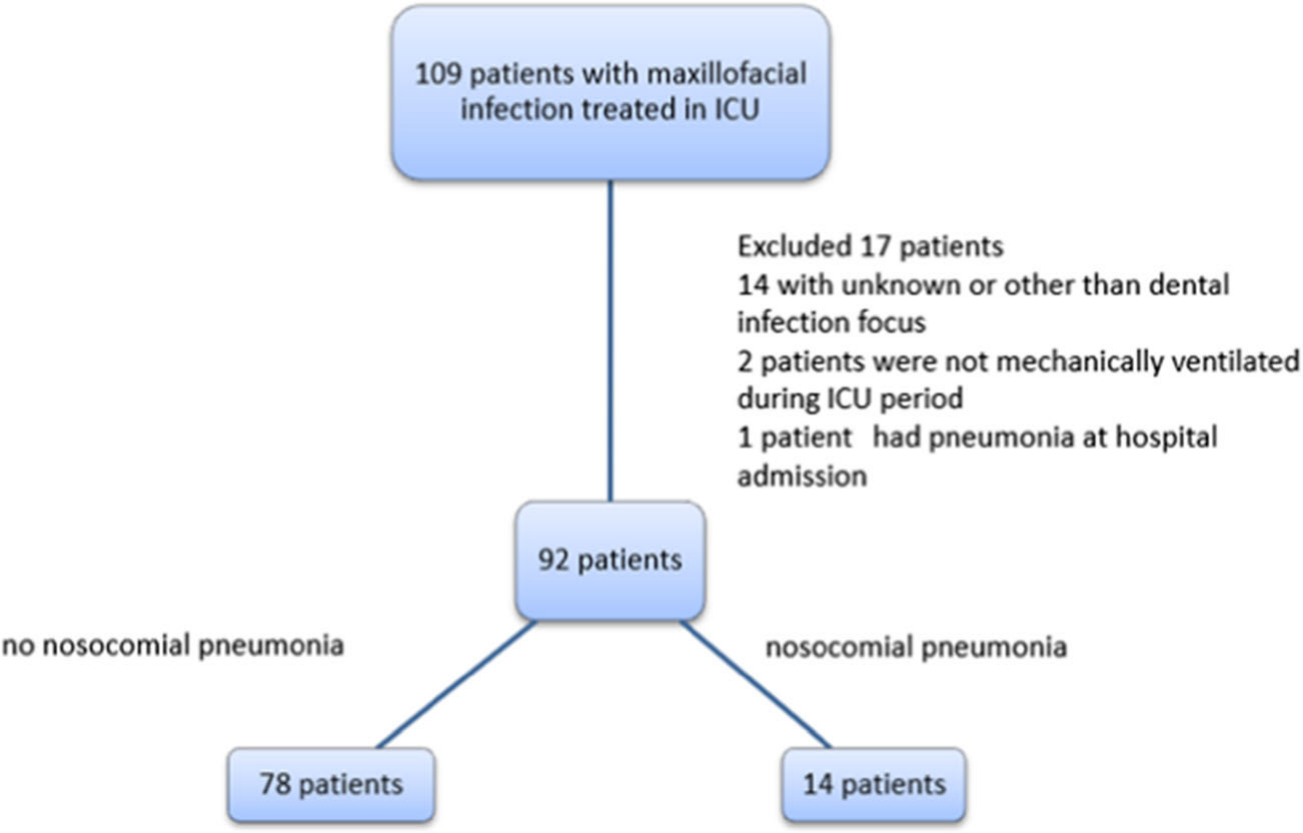
One hundred nine patients with maxillofacial infection treated in ICU

A total of 109 patients with oral and maxillofacial infection required treatment in ICU. Seventeen patients were excluded from the final analysis (Fig. 1). Thus, 92 patients remained to be analysed.

All 92 patients underwent surgical intervention of infection, i.e., incision and drainage of abscesses intra- and/or extra-orally and dental foci were identified and removed as a part of the surgical treatment if required. All patients were treated by oral and maxillofacial surgeons; thus, there was no delay in the focus identification and/or a focus tooth removal during hospital stay. One patient received tracheostomy at the primary stage of hospital care. The remaining 91 patients were intubated fiberoscopically or in a conventional manner. All options for airway management were available throughout the hospital stay. In addition to surgical treatment, all patients received antimicrobial medication (benzylpenicillin or cefuroxime combined with metronidazole or piperacillin combined with tazobactam).

Descriptive statistics of the 92 patients are presented in Table 1. Most of the patients were male (63 of 92, 68%). Patients’ age ranged from 18 to 88 years (mean 45 years, median 43 years). One-third of the patients were smokers (30 of 92, 33%). Of the patients, 9 out of 92 (10%) had DM, and 12 (13%) had a history of chronic pulmonary disease, which was in all cases asthma and/or chronic obstructive pulmonary disease (COPD). Eleven patients (12%) were heavy alcohol and/or drug abusers. LOHS ranged between 2 and 36 days (mean 7.3 days, median 6 days). Reintubation was required in 6 patients (7%). Interestingly the number of immunocompromised patients was very low in our study; in addition to DM patients, there were single patients suffering from pancreatic cancer, dilated cardiomyopathy, liver cirrhosis, and colitis ulcerosa.

**Table 1:** Descriptive statistics of 92 odontogenic infection patients requiring intensive care

Age (years)		
Range	18 to 88	
Mean	45	
Median	43	
	*n*	% of 92 patients
Gender		
Male	63	68
Female	29	32
Smoking		
Yes	30	33
No	62	67
Heavy alcohol and/or drug user		
Yes	11	12
No	81	88
Diabetes		
Yes	9	10
No	83	90
Chronic pulmonary disease		
Yes	12	13
No	80	87
Re-intubation		
Yes	6	7
No	86	93
Duration of ventilation treatment (days)		
Range	0 to 31	
Mean	4	
Median	3	
Length of hospital stay (days)		
Range	2 to 36	
Mean	7.3	
Median	6	

Pneumonia was clinically detected and radiologically confirmed in 14 of 92 patients (15%). It occurred between postoperative day 2 and 6 (mean 3 days, median 3 days). Four patients with pneumonia (29%) were diagnosed between 24 and 48 h of treatment at the ICU, and two of the patients had been extubated at the time of diagnosis. Diagnosis was confirmed with a chest x-ray in 13 patients and with computer tomography (CT) in one patient. Pneumonia diagnosis led to antibiotic medication change in 10 of 14 patients (71%). In all cases, antibiotic changes were made between beta-lactams; in one case clindamycin and in another case fluconazole was added to beta-lactams.

Associations between explanatory, predictor and additional variables and pneumonia are presented in Table 2 Pneumonia was more common in re-intubated patients (*p* = 0.004). Three out of four of these patients were diagnosed prior to re-intubation. Duration of mechanical ventilation (*p* = 0.028) and LOHS (*p* = 0.002) were significantly longer in patients with pneumonia. Pneumonia was significantly more common in smokers than in non-smokers (30% vs. 8%, *p* = 0.011), and the majority of patients with pneumonia were smokers (9 of 14, 64%).

**Table 2:** Associations between explanatory, predictor, and additional variables and pneumonia in 92 odontogenic infection patients

	Patients with pneumonia			Patients without pneumonia			
	*n*	%		*n*	%		*p* value
All	14	15		78	85		
Age (years)							
Range	21 to 79			18 to 88			
Mean	48			44			
Median	46			40			0.363
	*n*	%	% of patients with pneumonia	*n*	%	% of patients without pneumonia	
Gender							
Male	9	14	64	54	86	69	
Female	5	17	36	24	83	31	0.759
Smoking							
Yes	9	30	64	21	70	27	
No	5	8	36	57	92	73	0.011
Heavy alcohol and/or drug user							
Yes	1	9	7	10	91	13	
No	13	16	93	68	84	87	1.000
Diabetes	2	22	14	7	78	9	0.622
Asthma/COPD*	3	25	21	9	75	12	0.384
Re-intubation							
Yes	4	67	29	2	33	3	
No	10	12	71	76	88	97	0.004
Duration of ventilation treatment (days)							
Range	1 to 31			0 to 14			
Mean	8.5			3.1			
Median	6.5			2			0.028
Length of hospital stay (days)							
Range	4 to 31			2 to 36			
Mean	11.6			6.6			
Median	10			5.5			0.002

*COPD chronic obstructive pulmonary disease

In multivariate analysis with binomial logistic regression, our model was statistically significant, *χ*^2^ = 14.469, *p* = 0.043, and showed an excellent level of discrimination, with a value of 0.804 for the area under the receiver-operating characteristics curve and 0.254 for the Nagelkerke *R*^2^. Of the six predictor variables, only two were statistically significant: smoking and re-intubation, as shown in Table 3.

**Table 3:** Results of the binomial logistic regression model with occurrence of pneumonia as the dependent variable

Predictor variable	*B*	*p* value	OR	95% confidence interval
Gender, male	− 0.779	0.302	0.459	0.105–2.013
Age				
Lowest tertile (ref)				
Middle tertile	0.310	0.720	1.364	0.250–7.430
Highest tertile	0.262	0.754	1.300	0.252–6.705
Smoking	1.469	0.049	4.344	1.007–18.730
Re-intubation	2.196	0.038	8.990	1.135–71.178
Diabetes mellitus	0.871	0.415	2.390	0.294–19.431
Pulmonary disease	0.219	0.809	1.245	0.211–7.336

Eight patients received late tracheostomy during later ICU care; five of these patients had pneumonia (*p* = 0.002). In three of the five patients, pneumonia diagnosis was made prior to tracheostomy; however, none of the patients received tracheostomy principally because of pneumonia. All tracheostomies were performed due to prolonged ventilation by intubation and/or intensive swelling that was subsiding slower than anticipated.

Tracheal micro-organism aspirates were taken from 10 (71%) of 14 pneumonia patients, and in 9 cases, the micro-organism was identified in respiratory culture (Table 4). The same bacteria were detected in OI and tracheal aspirate only in one patient. The detected bacteria had no significant resistance to antibiotics, though it is not uncommon that bacteria found in aspirates of nosocomial pneumonia patients are resistant to common antibiotics.

**Table 4:** Comparison of odontogenic infection micro-organisms and tracheal aspirates in 9 patients

Micro-organism in odontogenic infection pus	Micro-organism in tracheal aspirate
*Streptococcus anginosus*, *Prevotella intermedia*	*Serratia marcescens*, *Candida dubliniensis*
Anaerobic gram + cocci	*Candida albicans*
Anaerobic gram + cocci	Respiratory tract normal flora
*Streptococcus anginosus*, *Prevotella intermedia*, anaerobic gram – rod	*Moraxella catarrhalis*
*Streptococcus anginosus*, *Candida albicans*, anaerobic mixed flora, *Actinomyces turicensis*	Respiratory tract normal flora, *Candida albicans*
Not taken	Coagulase-negative staphylococcus
Anaerobic gram – rods, oral normal flora	*Klebsiella oxytoca*, *Klebsiella pneumoniae* (gram – anaerobic rods)
*Streptococcus anginosus*, anaerobic mixed flora	*Streptococcus anginosus*, *Staphylococcus aureus*
*Streptococcus anginosus*, anaerobic mixed flora	*Staphylococcus aureus*

Overall mortality was 2% (2 of 92) during hospital care. Both deceased patients suffered from secondary pneumonia. Thus, 14% (2 of 14) of patients with pneumonia died during the ICU period following OI.

## Discussion

The purpose of this study was to clarify the occurrence and characteristics of pneumonia as a nosocomial infection in patients treated for deep OI. Our hypothesis was that pneumonia occurrence is rather high in OI patients in general and that treatment-related factors can be detected. The hypothesis was confirmed partially. Pneumonia occurred in no more than 15% of patients treated for deep OIs under general anaesthesia and requiring intensive care. Duration of mechanical ventilation (*p* = 0.028) and LOHS (*p* = 0.002) were significantly related to pneumonia, which are in concordance with previous observations. As a notable finding, smokers had a significant risk for nosocomial pneumonia (*p* = 0.011).

Studies focusing on OI patients’ nosocomial pneumonias are scarce, and occurrence of pneumonia has been shown to be moderate. In the study of Sittitrai et al. [2], pneumonia occurred in 5% of patients with deep neck infections; however, the study focused on immunosuppressive patients and included infections with other than odontogenic aetiologies as well. The study of Ylijoki et al. [14] reported a 1% rate of pneumonia in OI patients, but the number of patients requiring ICU treatment was low. In the recent study of Velhonoja et al. [9], 6% of patients with deep neck infection had pneumonia as an infection complication. The occurrence in the present study is higher (15%), which is explained by the inclusion criteria. We only included patients requiring postoperative mechanical ventilation. As mentioned earlier, ICU patients’ hospital-associated pneumonia prevalence is 5–40% in the general patient population with diverse reasons for intensive care [16]. Considering the fact that all patients in the present study had increased risk for pneumonia due to difficult airway management and deep oral and neck infections leading to ICU stay, it is surprising that pneumonia occurrence was not higher than presented. In addition, it is noteworthy that the same bacteria were detected in tracheal aspirate and odontogenic pus in only one out of nine patients for whom a sample was available (Table 4). All patients who died during hospital stay had pneumonia prior to death. Ventilation-associated pneumonia (VAP) was the most common infection and cause of death in trauma patients [21], and in a similar population, VAP mortality was reported to be 12% [17]. However, pneumonia in the present study is rather nosocomial than VAP; our results are in concordance with these previous results. The estimated mortality of VAP is around 10%, but with surgical ICU patients, it is higher [16]. All-cause mortality associated with VAP is reported to be as high as 50%, although the rate is questioned and previous studies from the nineties have reported conflicting results [16]. Therefore, the risk of pneumonia should be minimized.

Prolonged mechanical ventilation raises the risk for pneumonia [22, 23], the risk peaking between days 5 and 9 [16], and pneumonia patients have a distinctly longer ICU stay [16] and also LOHS [23]. Up to 63% of pneumonias develop within 48 h of mechanical ventilation [23]. Our results are in line with the earlier findings; duration of mechanical ventilation was markedly longer among pneumonic patients and was related to pneumonia (*p* = 0.028). In all patients, the reason for mechanical ventilation postoperatively was narrowed laryngeal space, and it was estimated in collaboration between oral and maxillofacial surgeons and anaesthesiologists. Previously, it has also been shown that re-intubation episodes predispose to pneumonia [19, 20, 22]. Although re-intubation rates were low in our study, it should be noted that 4 (67%) out of 6 re-intubated patients had pneumonia, and 29% of all pneumonia patients were re-intubated during ICU stay. However, it must be emphasized that in three out of four patients with pneumonia, it was diagnosed before re-intubation, and the remaining patient received a pneumonia diagnosis on the same day that re-intubation was performed. All six re-intubations were performed because of breathing difficulties caused by narrowed laryngeal space due to persistent infection or respiratory failure. These re-intubated patients had a severe, deep OI and a narrowed airway that often lead to pneumonia which developed before reintubation. In addition, pneumonia in these patients may also have contributed to the need for reintubation. Thus, pneumonia was not the result of re-intubation, but rather it occurred in patients with a constricted airway. Declining of infection, subsiding of swelling, and an open airway should be ensured prior to extubation to avoid the need for reinstitution of ventilatory support, especially in a population such as the present one in which intubation is often challenging. On the other hand, a longer mechanical ventilation period raises the risk of pneumonia, so effective early infection treatment and timing of extubation are crucial. Smooth communication between the ICU physician and the oral and maxillofacial surgeon is essential when making treatment decisions about extubation or continuation of mechanical ventilation.

Only one of the 92 patients had tracheostomy performed at the primary stage of OI treatment; this patient did not develop nosocomial pneumonia during the ICU period. Tracheostomy has been thought to decrease complications related to longer intubation times in patients with deep neck infection according to the study of Tapiovaara et al. [24]. In that study, patients were treated mostly for otolaryngological infections. In lower neck infections, tracheostomy may be considered, and patients can be transferred to the ward earlier. With regard to pneumonia, a randomized and controlled study in mechanically ventilated adult ICU patients found no difference in the incidence of VAP between early and late tracheostomy patients [25]. According to our results, routine tracheostomy in all OI patients requiring ICU treatment to prevent secondary pneumonia is unnecessary and would expose patients pointlessly to the disadvantages of tracheostomy. In smokers it may be advisable to consider early tracheostomy if patient’s clinical status is poor and duration of mechanical ventilation is expected to continue long.

In our study, pneumonia developed in 30% of smokers, while only 8% of non-smokers had pneumonia. In addition, almost two-thirds (64%) of pneumonia patients were smokers. In a recent publication by Du et al. [26], smoking was associated with a longer ICU period in spine trauma patients, but it was not found to increase the risk for pneumonia [26]. The authors stated that the small sample size may have caused a false-negative bias [26]. Smoking has also been found to correlate with the LOHS in patients with deep neck infections, thereby increasing the risk for complications [27]. Therefore, preventing the risk of pneumonia, especially in smokers, is warranted.

DM or previous chronic pulmonary disease did not predispose to pneumonia in the present study. Although studies of OI and pneumonia remain scarce, in different OI settings both DM and COPD have been identified as significant risk factors for nosocomial pulmonary infections, and patients with DM have been shown to have different and rarer bacterial strains and a significantly higher drug resistance rate than patients without DM [28–31]. In large cohorts, the pneumonia risk in DM patients seems obvious; López-de-Andrés et al. [32] reported a 21% higher incidence of postoperative pneumonia in DM patients than in non-diabetic patients in a cohort of over 100,000 patients, although contradictory results have also been reported [33, 34]. Associations between chronic pulmonary disease or DM and pneumonia were not verified in our study; however, the number of patients with a history of these diseases was too low for reliable statistical evaluation.

Interestingly, none of the pneumonia patients were iatrogenically immunosuppressed. One pneumonia patient had labile DM type 1, which predisposes to infections in general, one patient had DM type 2, and one patient had dilated cardiomyopathy. The present findings that immunosuppressive patients were not exposed to pneumonia and that patients had low susceptibility to infections in general may be due to effective treatment in early infections. It is a common practice that all dental infections must be treated before any immunosuppressive medication (i.e., biological medications or cytostatic treatment) is started. In addition, patients are informed that dental hygiene and treatment are important during use of these medications.

Prevention of nosocomial pneumonia in mechanically ventilated patients is crucial. The best way to prevent VAP is to critically assess the need for mechanical ventilation and evaluate daily the conditions for extubation. According to our hospital protocol, it is recommended to use as light sedation as possible, the head of the bed should be elevated to prevent micro-aspiration of stomach contents, and the collection of secretion above the cuff must be minimized and the cuff pressure checked and optimized to 20–30 cm H_2_O. Careful oral care combined with use of mouth rinse, such as hydrogen peroxide [35], is a local and easy treatment method and is particularly well-suited for OIs to prevent colonization of oral pathogens on the endotracheal tube. The use of oral chlorhexidine (CHX) is controversial and unclear in preventing pneumonia. The study of Papazian et al. [16] noted that the use of oral CHX may not be recommended because no association has been shown between CHX use in oral care and lower rates of pneumonia; on the contrary, it seems to increase mortality rates perhaps due to aspiration. Then again, a systematic review by Hua [36] has shown that the use of oral CHX decreases the risk of VAP from 25 to 19%.

In the present study, clinical suspicion of pneumonia was verified by chest x-ray in 13 patients and by CT in one patient. The determination of pneumonia in patients treated for another severe infection in ICU is challenging, and diagnosis based solely on imaging is not reliable [37]. ICU patients’ radiographs are most often performed in a lying position, rendering interpretation of the image more difficult. It is known that chest x-ray is not sensitive or specific in VAP diagnostics; in the study of Papazian et al. [16], two patients’ chest x-rays were falsely negative. More precise criteria than clinical suspicion and radiological confirmation of pneumonia were not used; therefore, misdiagnosis or incorrect diagnosis of pneumonia may have occurred. Thus, this can be considered a clear weakness of the study. In addition, data of possible comorbidity of the pneumonia patients, assessment of lung function capacity, as well as details of mechanical ventilation would have clarified the risks of VAP more. In deep OIs, the intubation procedure is usually challenging due to swelling, and there is also a possibility of aspiration of, e.g. bursting pus. We were unable to analyse these factors due to the retrospective nature of the study. To clarify more the risk of pneumonia in OI patients, more extensive patient data including tracheal aspirates, evaluation of intubation circumstances, and more detailed data of ICU period would be required.

## Conclusion

Overall, 15% of all OI patients treated in ICU with mechanical ventilation had nosocomial pneumonia. The study shows that challenging airway circumstances, prolonged restricted airway, lengthy mechanical ventilation, and smoking predispose to nosocomial pneumonia in the OI population. The risk of pneumonia can be reduced by early and prompt treatment of OI, thereby decreasing the duration of mechanical ventilation.
